# The viral capping enzyme nsP1: a novel target for the inhibition of chikungunya virus infection

**DOI:** 10.1038/srep31819

**Published:** 2016-08-22

**Authors:** L. Delang, C. Li, A. Tas, G. Quérat, I. C. Albulescu, T. De Burghgraeve, N. A. Segura Guerrero, A. Gigante, G. Piorkowski, E. Decroly, D. Jochmans, B. Canard, E. J. Snijder, M. J. Pérez-Pérez, M. J. van Hemert, B. Coutard, P. Leyssen, J. Neyts

**Affiliations:** 1 KU Leuven – University of Leuven, Department of Microbiology and Immunology, Rega Institute for Medical Research, Laboratory of Virology and Chemotherapy, Minderbroedersstraat 10, 3000 Leuven, Belgium; 2 CNRS, AFMB UMR 7257, Avenue de Luminy 163, 13288 Marseille, France; 3 Aix-Marseille Université, AFMB UMR 7257, Avenue de Luminy 163, 13288 Marseille, France; 4 Molecular Virology Laboratory, Department of Medical Microbiology, Leiden University Medical Center, Albinusdreef 2, 2333 ZA Leiden, The Netherlands; 5 Aix-Marseille Université, IRD French Institute of Research for Development, UMR_D 190 “Emergence des Pathologies Virales”, 27 boulevard Jean-Moulin, 13005 Marseille, France; 6 Instituto de Química Médica, Consejo Superior de Investigaciones Científicas (IQM-CSIC), Juan de la Cierva 3, 28006 Madrid, Spain

## Abstract

The chikungunya virus (CHIKV) has become a substantial global health threat due to its massive re-emergence, the considerable disease burden and the lack of vaccines or therapeutics. We discovered a novel class of small molecules ([1,2,3]triazolo[4,5-*d*]pyrimidin-7(6*H*)-ones) with potent *in vitro* activity against CHIKV isolates from different geographical regions. Drug-resistant variants were selected and these carried a P34S substitution in non-structural protein 1 (nsP1), the main enzyme involved in alphavirus RNA capping. Biochemical assays using nsP1 of the related Venezuelan equine encephalitis virus revealed that the compounds specifically inhibit the guanylylation of nsP1. This is, to the best of our knowledge, the first report demonstrating that the alphavirus capping machinery is an excellent antiviral drug target. Considering the lack of options to treat CHIKV infections, this series of compounds with their unique (alphavirus-specific) target offers promise for the development of therapy for CHIKV infections.

Chikungunya virus (CHIKV) is an arthropod-borne virus that is primarily transmitted by *Aedes aegypti* and *Aedes albopictus* mosquitoes. Before 2004 only sporadic and relatively limited outbreaks were reported, but later large-scale outbreaks have occurred in many tropical regions in Africa and Asia^1^. Concomitantly, multiple imported cases among travelers returning from endemic areas have been reported in several European countries, the USA, Canada and Australia. Because of the spread of in particular the *Aedes albopictus* mosquito to more temperate regions such as Southern Europe, Northern Asia and the Americas, it was anticipated that CHIKV has the potential to become endemic in new regions^2^,^3^. In December 2013, the first locally transmitted infections were reported in the Americas on the Caribbean island of Saint Martin. From there, the virus has been spreading further to neighboring countries in the Caribbean and South as well as Central America^4^. Over 1.6 million cases have been reported till October 2015 in the Americas by the WHO/PAHO. Severe and fatal acute CHIKV infections have also been described during this continuing epidemic^5^,^6^.

Chikungunya fever is mostly characterized by a very painful arthralgia that usually resolves within several days^7^. Although CHIKV infections are rarely fatal, up to 60% of the patients develop a chronic disease that is characterized by persistent and often disabling polyarthritis, which can severely incapacitate the patient for weeks up to several years after the acute infection^8^. Due to the severe symptoms and its re-emergence on a massive scale, CHIKV has become a substantial public health problem. There is no approved vaccine or antiviral drug available for the prevention or treatment of this infection. Patients are currently given analgesics, antipyretics and anti-inflammatory agents to alleviate their symptoms. Chloroquine, a drug that is commonly used for the treatment of malaria, was demonstrated to have a dose- and time-dependent inhibitory effect on CHIKV replication *in vitro*^9^, but clinical studies failed to prove its efficacy in infected patients^10^,^11^. We demonstrated that Favipiravir (T-705, approved in Japan for the treatment of influenza infections) exerts anti-CHIKV activity *in vitro* and in a mouse model^12^. Several other molecules with *in vitro* anti-CHIKV activity have been reported^13^, but to our knowledge, none of these molecules have progressed towards further development.

We recently identified - in a large scale cell-based antiviral screening campaign - 3-(3′-acetylphenyl)-5-methyl-3*H*-[1,2,3]triazolo[4,5-*d*]pyrimidin-7(6*H*)-one (MADTP-314) as a selective inhibitor of CHIKV replication. Following initial hit-optimization more potent analogs were obtained^14^. We here report on the unique molecular mechanism by which these compounds inhibit CHIKV replication, through targeting the alphavirus capping machinery.

## Results

### Antiviral activity of the MADTP-series against chikungunya virus and other alphaviruses

**Figure 1:**
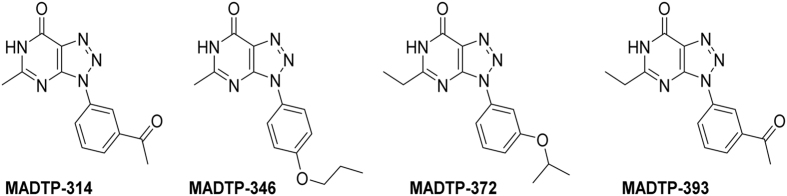
Chemical structures of MADTP-314, MADTP-346, MADTP-372 and MADTP-393.

Based on the initial hit MADTP-314 (Fig. 1), several series of MADTP compounds were synthesized and their efficacy was tested against the laboratory-adapted CHIKV strain 899^14^. The most potent and selective analog in this series identified so far, 5-ethyl-3-(3-isopropoxyphenyl)-3H-[1,2,3]triazolo[4,5-d]-pyrimidin-7(6H)-one (MADTP-372, Fig. 1), inhibited the induction of the cytopathic effect (CPE) by CHIKV with an EC_50_ value of 2.6 μM (CC_50_ > 400 μM) (Table 1)^14^. Combining the ethyl substituent at position 5 of the heterocyclic base in MADTP-372 and the 3′-acetyl group at the aryl moiety present in the initial hit (MADTP-314) led to the synthesis of MADTP-393 (the synthesis of this molecule will be published elsewhere). This MADTP analog had a better anti-CHIKV profile than MADTP-314, but was not as potent as MADTP-372 (Table 1). MADTP-346 is a close structural analog that was devoid of anti-CHIKV activity.

The antiviral activity of selected MADTP analogues was next validated by quantifying the release of viral RNA in the supernatant by qRT-PCR. The MADTP-series proved active against clinical isolates of CHIKV, with the Congo 95 strain (clinical isolate of 2011) being the most susceptible [as shown for MADTP-314, -372 and -393 (Table 1)]. No or modest antiviral activity was observed against other alphaviruses such as the Sindbis virus, the Semliki forest virus, the O’Nyong Nyong virus, the Barmah forest virus, the Mayaro virus and the Ross River virus (data not shown). Only MADTP-372 significantly inhibited the replication of Venezuelan equine encephalitis virus (VEEV) in cell culture (Table 1).

### The mechanism of action of MADTP-314

**Figure 2:**
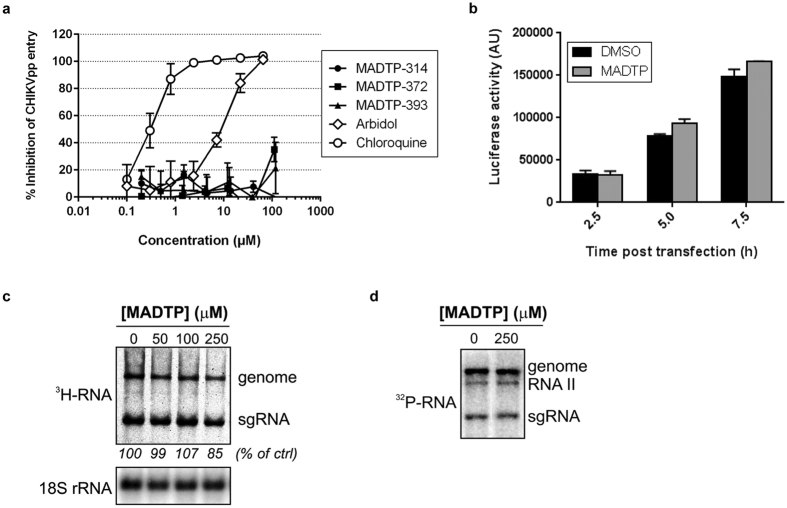
Mechanism of action of the MADTP series. (**a**) Huh 7.5.1 cells treated with different MADTP compounds were infected with CHIKV pseudoparticles. Arbidol and chloroquine were used as positive controls. The entry of CHIKVpp was determined by measuring the luciferase activity. The average values ± SD of three independent experiments are shown. (**b**) Cells were transfected with a replication-deficient CHIKV RNA encoding an nsP3-Rluc fusion protein, in the presence or absence of MADTP-314. Subsequently, luciferase activity was determined at 2.5, 5 and 7.5 h post-transfection. (**c**) CHIKV-infected Vero E6 cells (MOI 5), treated with 50, 100 and 250 μM MADTP-314, were metabolically labeled with ^3^H-uridine from 6–7 h p.i. Total RNA was isolated and separated by denaturing agarose gel electrophoresis, followed by fluorographic detection of ^3^H-labeled RNA. (**d**) *In vitro* RNA synthesizing activity of isolated CHIKV RTCs in the presence or absence of 250 μM MADTP-314, quantified by measuring the incorporation of ^32^P-CTP. RNA II is a 7.5 kb CHIKV RNA that corresponds to the 5′-proximal 7.5 kb of the CHIKV genome up to the subgenomic promoter region^17^.

MADTP-314 was selected (at the time of the ongoing hit-optimization process the most potent/selective inhibitor in the series) as the prototype of this class of molecules to study the particular characteristics and the mechanism of action of its anti-CHIKV activity. Time-of-addition studies revealed that MADTP-314 had an inhibition profile that was comparable to that of a polymerase-targeting compound (T-705^12^), i.e. the compound remained active when addition to the infected cultures was delayed by several hours (up to 6 h) post infection (1.3 log_10_ and 1.0 log_10_ reduction in CHIKV RNA for MADTP-314 and T-705, respectively, when added 6h post infection and assessed at 24 h post infection). In contrast, chloroquine, a CHIKV entry inhibitor included as a reference compound, entirely lost its protective activity when added at time points later than 1 h p.i., whereas a 2 log_10_ reduction of CHIKV RNA was obtained when the compound was added 2 h before infection. To study whether MADTP-314 can block CHIKV cell entry, CHIKV pseudoparticles (CHIKVpp) were produced as described before^15^. These pseudoparticles are lentiviral vectors carrying CHIKV glycoproteins and are thus ideally suited to study CHIKV entry independently from post entry steps in the viral life cycle. In line with the time-of-addition experiments, MADTP-314 and analogs were not able to block the entry of CHIKV pseudoparticles (CHIKVpp) into the host cell (Fig. 2a). In contrast, CHIKV entry inhibitors such as arbidol and chloroquine inhibited CHIKVpp entry in a dose-dependent manner (Fig. 2a)^9^,^16^. To assess whether MADTP-314 (directly) affects translation of incoming capped CHIKV RNA genomes, cells were transfected with a replication-deficient CHIKV RNA (carrying an active site mutation in the RNA polymerase domain of nsP4) encoding an nsP3-luciferase fusion protein, which allowed measuring translation independent of RNA replication. Luciferase activity in cells treated with 250 μM MADTP-314 was comparable to that in untreated control cells, suggesting that the compound did not directly inhibit translation of the incoming, capped genomic RNA (Fig. 2b). Next we assessed whether MADTP-314 had a direct inhibitory effect on CHIKV RNA synthesis as quantified by measuring ^3^H-uridine incorporation into newly synthesized CHIKV RNA. In CHIKV-infected cells, MADTP-314 (50–250 μM) did not inhibit CHIKV RNA synthesis (Fig. 2c). This was confirmed in an *in vitro* assay with isolated replication/transcription complexes (RTCs)^17^, which demonstrated that 250 μM of MADTP-314 did not inhibit the incorporation of ^32^P-CTP into CHIKV RNA (Fig. 2d). Taken together these results demonstrate that MADTP-314 inhibits CHIKV replication at a post-entry step, other than translation or viral RNA synthesis.

### Selection and characterization of MADTP-314-resistant CHIKV variants

To identify the viral protein that is targeted by the MADTP compounds, CHIKV variants with resistance to MADTP-314 were selected. To this end a 5-step resistance selection protocol was used as described before^12^. Three MADTP-314-resistant virus isolates were independently obtained from a heterogeneous (quasi-species) wild-type population of CHIKV (strain 899). All three variants proved ~11-fold less sensitive to the antiviral effect of the compound (Table 2). The selected resistant variants were cross-resistant to the antiviral effect of other MADTP analogs. No cross-resistance to chloroquine or T-705 was observed (Table 2), suggesting a different mechanism of action for the MADTP compounds.

A total of five mutations were identified in the 3 variants by means of population sequencing, including a mutation in the nsP1 gene (Supplemental Table 1). This P34S substitution in nsP1 was the only amino acid change that all three of the MADTP-314-resistant isolates had in common. Interestingly, P34 is located near a conserved histidine at position 37 in a region of nsP1 known to contain several key residues for the mRNA capping functions (Supplemental Figure 1A,B)^18^.

### Involvement of the P34S substitution in the MADTP-resistant phenotype

**Figure 3:**
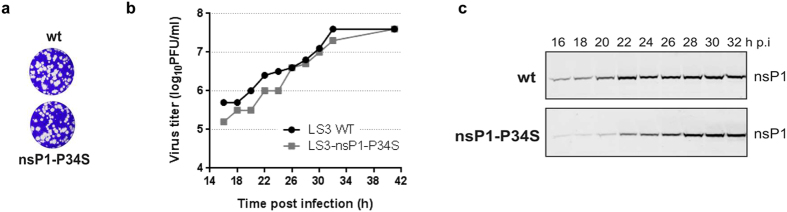
Characterization of the CHIKV LS3-nsP1-P34S mutant. (**a**) Plaque morphology of CHIKV LS3 and mutant CHIKV LS3-nsP1-P34S at 3 days post infection. The plaque assay was performed on Vero E6 cells as described in ref. 19. (**b**) Growth curve of CHIKV LS3 (black) and mutant CHIKV LS3-nsP1-P34S (grey) on Vero E6 cells (MOI 0.05). Virus titers were determined by plaque assay on Vero E6 cells at 3 days p.i. Data shown are average values of two independent experiments. (**c**) The expression of nsP1 in Vero E6 cells infected with WT CHIKV LS3 or the nsP1-P34S mutant virus at various time points post infection (MOI 0.05) analyzed by Western blot.

The mutation that causes the P34S substitution in nsP1 was introduced into infectious clone CHIKV LS3^19^ by reverse genetics. Serial passaging on Vero E6 cells in the absence of MADTP and sequencing of the virus revealed that the mutation was stable for at least 5 passages and did not markedly affect viral fitness. Indeed plaque morphologies of CHIKV-LS3-nsP1-P34S and WT CHIKV-LS3 were comparable (Fig. 3a) and the P34S mutant replicated only slightly slower than WT in growth curve experiments (Fig. 3b). Western blot analysis of CHIKV‐infected Vero E6 cells at various time points p.i. indicated that the accumulation of nsP1 in cells infected with the P34S mutant virus was somewhat reduced compared to WT (Fig. 3c). The P34S mutation thus seems to only modestly affect the growth characteristics of the virus.

**Figure 4:**
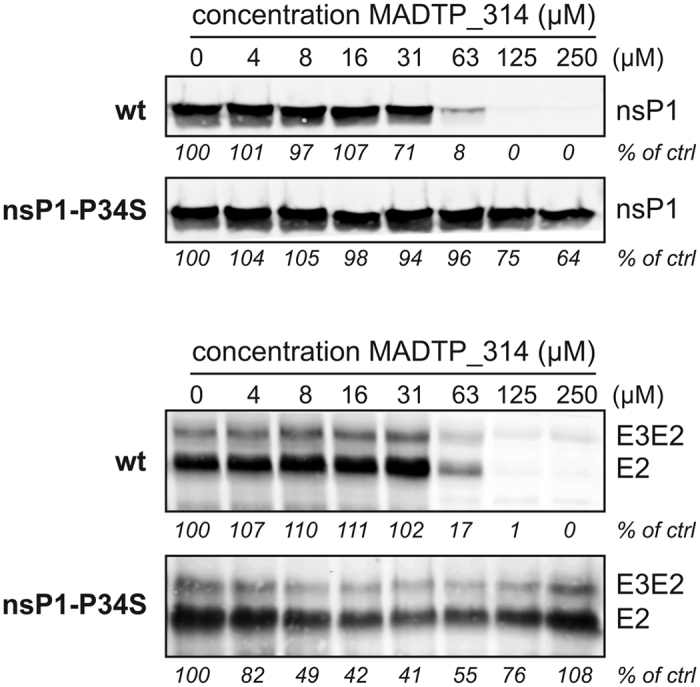
Sensitivity of WT and nsP1-P34S mutant virus to MADTP-314. Effect of various concentrations of MADTP-314 on the expression of nsP1 and the structural protein E2 of WT CHIKV LS3 and the P34S-LS3 mutant CHIKV in infected Vero E6 cells (MOI 0.1) that were analyzed by Western blotting at 28 h p.i. Percentages of untreated control are presented below the blots. The anti-E2 antibody was a gift of Dr. G. Pijlman^34^, the anti-nsP1 antibody was published before in ref. 33.

Next, using a CPE-based antiviral assay, the sensitivity to MADTP-314, MADTP-372, and MADTP-393 was determined for CHIKV-LS3-nsP1-P34S and the parental WT CHIKV-LS3 (Table 2). In contrast to the WT virus, the reverse-engineered mutant nsP1-P34S virus proved to be highly resistant to the antiviral effect of the three different MADTP compounds [it should be noted that CHIKV strain LS3 was 1.5-fold less sensitive to MADTP-314 than CHIKV 899]. By analyzing CHIKV protein expression in infected cells, the nsP1-P43S mutant virus was also shown to be highly resistant to MADTP-314, as nsP1 and E2E3 proteins were easily detected in cells treated with MADTP-314 concentrations above 63 μM, whereas such treatment resulted in hardly detectable expression of CHIKV proteins in cells infected with the WT virus (Fig. 4). In conclusion, these results clearly linked the nsP1-P34S mutation to phenotypic resistance to MADTP compounds.

### Effect of MADTP molecules on the alphavirus capping machinery

The alphavirus nsP1 protein possesses both methyltransferase (MTase) and nsP1 guanylyltransferase (GT) activities. The protein catalyzes the transfer of the methyl group from S-adenosylmethionine (AdoMet) to the N7 position of a GTP molecule, which is followed by the formation of an m^7^GMP-nsP1 adduct. The subsequent transfer of m^7^GMP onto the 5′ end of the viral RNA has been recently demonstrated *in vitro* for VEEV nsP1^18^. To explore the potential effect of the MADTP-series on the *in vitro* enzymatic activity of nsP1, we extensively attempted to purify recombinant CHIKV nsP1 from *E. coli*, but unfortunately failed to obtain enzymatically active protein. Since *in vitro* assays were available for purified VEEV nsP1, we assessed the anti-VEEV activity of the MADTP compounds and found that MADTP-372 caused a significant inhibition of VEEV replication in cell culture (Table 1, EC_50_ of 6.8 μM). We therefore decided to assess the effect of this compound on the enzymatic activities of VEEV nsP1 using the available biochemical assays. The results of the expression, purification and GTase activity of both CHIKV and VEEV nsP1 are shown in Supplemental Figure 2.

**Figure 5:**
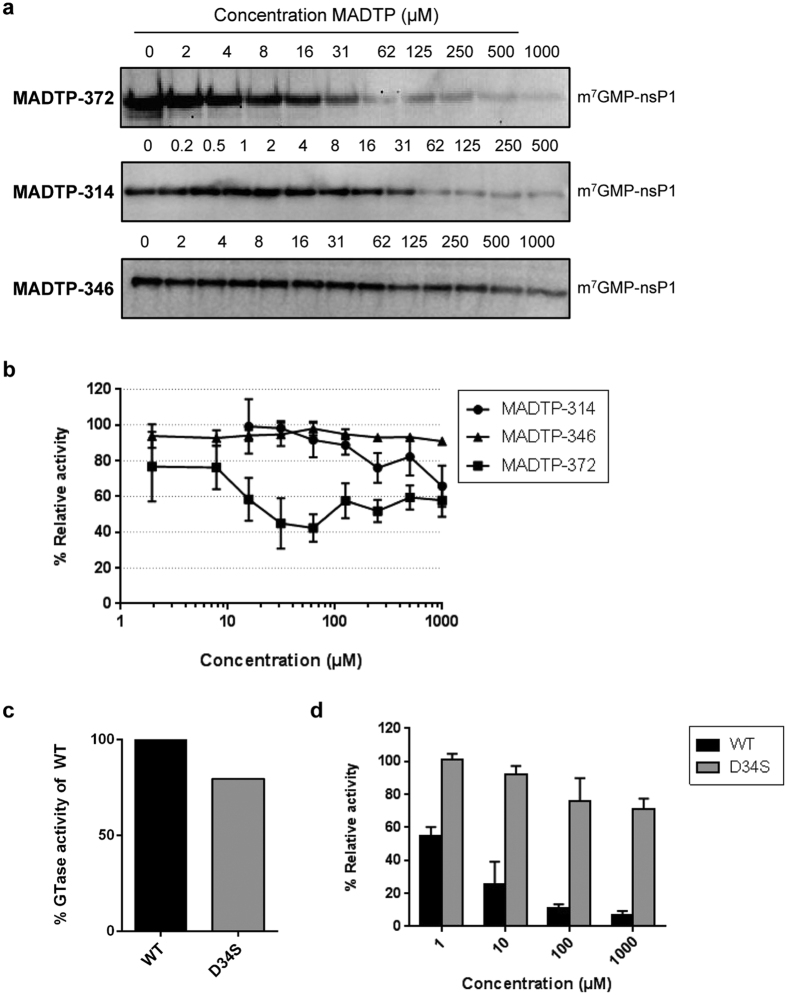
Effect of MADTP compounds on the methyltransferase and the guanylyltransferase activity of VEEV nsP1. (**a**) Effect of different concentrations of MADTP-372, MADTP-314 and MADTP-346 on the *in vitro* guanylylation of VEEV nsP1. The m^7^GMP-nsP1 complex was detected by Western blot with an anti-methyl^3/7Gp^ antibody. (**b**) Dose-response curves of MADTP-314 (circles), MADTP-346 (triangles), and MADTP-372 (squares) on the methyltransferase activity of VEEV nsP1. The nsP1-MTase product (3H-methyl) GIDP was measured by a scintillation counter. The average values ± SD of two independent experiments are shown. (**c**) Relative nsP1 guanylylation activity (% WT) of the D34S-nsP1 mutant in the absence of compounds. (**d**) Relative nsP1 guanylylation activity (% untreated control) of WT and D34S VEEV nsP1 when treated with different concentrations of MADTP-372. The average values ± SD of two independent experiments are shown.

The *in vitro* guanylyltransferase activity of VEEV nsP1 was quantified by measuring the formation of the m^7^GMP-nsP1 complex by Western blotting, using m^7^GTP as a substrate and an anti-methyl^3/7Gp^ antibody for detection. MADTP-372, and to a lesser extent MADTP-314, inhibited the GT activity of VEEV nsP1 in a concentration-dependent manner (Fig. 5a). In contrast, MADTP-346, an analog devoid of antiviral activity in cell culture, did not inhibit the GT activity of VEEV nsP1. To further characterize the mode of action of MADTP compounds, the MTase activity of VEEV nsP1 was quantified by a filter-based assay using ^3^H-AdoMet. MADTP-314, MADTP-346 and MADTP-372 did not or only modestly inhibit the MTase activity of VEEV nsP1 (IC_50_ > 1000 μM) (Fig. 5b).

### Resistance of VEEV nsP1-D34S to MADTP-372

To assess the role of the amino acid at position 34 in the molecular mechanism of the antiviral resistance against the MADTP series, D34 (corresponding to CHIKV P34) of VEEV nsP1 was mutated to a serine. The GT activity of the recombinant VEEV nsP1 was barely affected by the D34S mutation (Fig. 5c). Thus, the inhibitory effect of MADTP-372 on the GT reaction could be assessed for both the WT and the mutated protein. The D34S mutation alleviated the inhibitory effect of the compound on the GT reaction with IC_50_ values increasing from 10 ± 0.3 μM for WT to ~1000 μM for the mutant protein (Fig. 5d). These biochemical assays on WT and mutant D34S VEEV nsP1 thus confirmed that the MADTPs target the enzymatic activity of nsP1.

## Discussion

Many RNA viruses have adopted a strategy to modify the 5′ end of their genome by the covalent attachment of a peptide or the introduction of a cap moiety to ensure efficient translation, to protect their RNA from degradation by cellular 5′-3′ exonucleases, and to avoid detection as foreign RNA (which can trigger an innate immune response)^20^. Through a non-conventional cytoplasmic capping mechanism, alphaviruses synthesize the minimal RNA cap chemical structure, named cap-0 (m^7^GpppNp-RNA, in which N is the first nucleotide of the viral RNA, p represents a phosphate and m represents a methyl group)^20^. Several reactions are required for the synthesis of the alphavirus cap structure. The N7 position of GTP is first methylated by the methyltransferase activity of nsP1 using S-adenosylmethionine as a methyl-donor. Next a covalent m^7^GMP-nsP1 complex is formed from which the m^7^GMP is transferred to the viral RNA. The amino acid binding covalently to m^7^GMP has never been characterized directly, but histidine 37 has been proposed as the best candidate^18^,^21^,^22^. Before guanylyltransfer to the viral RNA can occur, the 5′ gamma-phosphate of the nascent viral RNA chain is removed by an RNA 5′-triphosphatase residing in the nsP2 protein^23^,^24^.

Since RNA capping is an essential step in the replication cycle of alphaviruses (and many other viruses) and since it differs from the host cell’s mechanism by its localization and reaction sequence, this process may be a potential target for antiviral drug development. For instance, the cap-snatching mechanism by which orthomyxoviruses acquire their cap has been shown to be a good target for inhibitors of influenza virus replication^25^. Only a few selective inhibitors of viral RNA cap formation have been reported till now^26^, and to the best of our knowledge, none of them affect alphavirus capping. Inhibitors of viral capping that were previously reported target the MTases of flaviviruses and coronaviruses^26^. However, these MTase inhibitors mostly encompass GTP and S-adenosylmethionine/homocysteine analogs^27^, which raises concerns about their specificity and potential toxicity (through their effect on host MTases). For alphaviruses, the capping mechanism driven by the multifunctional nsP1 protein could be an attractive target for antiviral treatment since it differs from its cellular counterpart. However, the fact that no structure of alphavirus nsP1 proteins have been reported has hampered the development of capping inhibitors by structure-based drug design^28^.

We here demonstrate for the first time that small molecule inhibitors of the alphavirus nsP1 RNA capping machinery are able to efficiently and completely abolish CHIKV replication *in vitro* at concentrations that have no adverse effect on the host cell. As shown with the prototype compound MADTP-314, these triazolopyrimidinones do not inhibit CHIKV entry, which was corroborated by time-of-drug-addition experiments. Transfection of cells with replication-incompetent CHIKV RNA revealed that MADTP-314 did not directly affect the translation of incoming capped genomes, demonstrating that there is no cap-independent modulation of viral protein synthesis. The compound also had no direct effect on CHIKV RNA synthesis. Compound-resistant virus variants were selected that carried the P34S mutation in the nsP1 protein and reverse genetics confirmed that this mutation is responsible for resistance to MADTP-314.

To explore the effect of this class of compounds on the enzymatic functions of the CHIKV nsP1 protein, we set out to express and purify this protein. However, despite dedicated efforts, we were not able to obtain enzymatically active CHIKV nsP1. Interestingly, MADTP-372, a close structural analog of MADTP-314, also inhibited VEEV replication in cell culture. The anti-VEEV effect of MADTP-372 led us to assess the effect of this compound on the enzymatic activity of the VEEV nsP1, for which we previously succeeded to obtain enzymatically active recombinant protein^18^. A clear dose-dependent inhibition of the guanylyltransfer (GT) activity was observed in a Western blot-based assay. MADTP-314 also showed a modest inhibitory effect on the GTase activity, while MADTP-346, an analog devoid of anti-CHIKV activity in cell culture^14^, proved virtually inactive against the GT activity of purified VEEV nsP1. So far we were unable to select MADTP-resistant VEEV variants.

Previous sequence analysis and functional studies have shown that the sequence surrounding amino acid 34 of nsP1, which is mutated in MADTP-resistant variants, is a key domain for alphavirus mRNA cap synthesis^18^. It is located at the beginning of the core region that carries both MTase and GT activities^28^. More precisely, nsP1 H37, the amino acid that is the putative acceptor for the guanylylation, is in close proximity to P34. At this stage, in the absence of information on the structure of alphavirus nsP1, it would be tempting to propose that MADPTs inhibit the GT reaction by hampering either the binding of m^7^GTP or the catalysis of the guanylylation itself. However, other possible scenarios that involve indirect effects cannot be ruled out.

It is intriguing that, despite the well conserved, enzymatically active part of alphavirus nsP1, the reported series of compounds does not inhibit closely related alphaviruses like ONNV. It could be that some alphaviruses have mechanisms to overcome defects in cap-synthesis activity. It was also shown recently that SINV produces non-capped viral genomic RNAs, especially in significant numbers during the early phase of infection^29^. It is not clear whether this is also the case for CHIKV or other alphaviruses. It may thus be that CHIKV does not have this mechanism and that it is therefore very sensitive to nsP1 inhibitors. The optimization of this compound series is still ongoing and we observed that the second generation of compounds has a modest antiviral activity against ONNV (EC_50_ values between 15–25 μM).

As there are currently no options to treat infections with the re-emerging CHIKV, the MADTP compounds with their unique (alphavirus-specific) target provide a promising starting point to develop therapy for CHIKV infections (and for which the classical preclinical and clinical studies need to be carried out). In such case that the structure of the nsP1 of CHIKV or of another alphavirus would be resolved in the future, this could enable structure-based drug design of new classes of capping inhibitors. It remains to be studied (first in animal models) whether or not CHIKV inhibitors may have a beneficial effect on chronic infections of the joints with this virus. If this were to be the case, this will offer important options for the management of this debilitating condition. Potent and safe CHIKV inhibiting drugs may also be used prophylactically, for example in travelers to endemic regions or as household prophylaxis.

In conclusion, we here report for the first time on a class of small-molecule inhibitors of CHIKV replication that block the guanylyltransferase activity of nsP1, thereby preventing the formation of capped viral mRNA. We thus provide proof that the (alpha)viral RNA capping machinery is a druggable target for the development of desperately needed, selective and potent antiviral therapy against CHIKV.

## Methods

### Cells and virus strains

CHIKV Indian Ocean strain 899 (Genbank FJ959103.1) was generously provided by Prof. C. Drosten (University of Bonn, Germany)^30^. The clinical isolates Venturini and Bianchi (Italy 2008) and Congo 95 (2011) belong to the collection of viruses at the UMR 190, Marseille, France, as well as O’Nyong Nyong virus strain IPD A234, Mayaro virus strain TC625, Barmah Forest virus strain BH2193, VEEV vaccine strain TC83. Ross River virus 5281v was received from the National Collection of Pathogenic Viruses (UK). CHIKV LS3 (GenBank KC149888) that was used for reverse genetics studies is derived from an infectious cDNA clone belonging to the collection of the Leiden University Medical Center, The Netherlands^19^. Sindbis virus (SINV, strain HRsp, GenBank J02363.1) and the Semliki Forest virus (SFV, Vietnam strain, GenBank EU350586.1^31^) belong to the collection of viruses at the Rega Institute of Medical Research, Belgium. All viruses were propagated in African green monkey kidney cells [Vero cells (ATCC CCL-81)].

Vero A cells were maintained in cell growth medium composed of minimum essential medium (MEM Rega-3) supplemented with 10% Foetal Bovine Serum (FBS), 1% l-glutamine, and 1% sodium bicarbonate. Vero E6 cells were maintained in Eagle MEM supplemented with non-essential amino acids and 7.5% FBS. Huh 7.5.1 cells were maintained in Dulbecco’s modified essential medium (DMEM) supplemented with 10% FBS, 1% HEPES, 1% non-essential amino acids and 1% penicillin-streptomycin. The antiviral assays were performed in virus growth medium which is the respective cell growth medium supplemented with 2% (instead of 10%) FBS. All cell cultures were maintained at 37 °C in an atmosphere of 5% CO_2_ and 95–99% humidity.

### Compounds

The synthesis of MADTP-314, MADTP-346 and MADTP-372 was described previously^14^. The synthesis of MADTP-393 will be reported elsewhere. The structures of the compounds are depicted in Fig. 1. Compounds were dissolved in analytical grade dimethyl sulfoxide (DMSO). T-705 (favipiravir) was purchased as a custom synthesis product from BOC Sciences. Chloroquine was purchased from Sigma and dissolved in PBS. Arbidol was a kind gift of Prof. S. Polyak (University of Washington, Seattle, USA).

### Antiviral assays

Vero A cells were seeded in 96-well tissue culture plates at a density of 2.5 × 10^4^ cells/well in 100 μl assay medium and were allowed to adhere overnight. Next, a compound dilution series was prepared after which the cultures were infected with 0.001 MOI of CHIKV 899 inoculum in 100 μl assay medium. On day 5 post-infection (p.i.), the plates were processed using the MTS/PMS method as described by the manufacturer (Promega). The 50% effective concentration (EC_50_) was determined using logarithmic interpolation. Potential cytotoxic/cytostatic effects of the compound as well as the longevity of Vero cells were evaluated in uninfected cells by means of the MTS/PMS method. The 50% cytotoxic concentration (CC_50_) was calculated using logarithmic interpolation. All assay wells were checked microscopically for minor signs of virus-induced CPE or possible alterations to the cell or monolayer morphology caused by the compound.

A variant of this protocol was used for the reverse-engineered CHIKV LS3 virus. Briefly, 1 × 10^4^ Vero E6 cells were seeded per well in a 96-well tissue culture plate and infected the next day at an MOI of 0.001. On day 4 p.i., the plates were processed using the MTS/PMS method. EC_50_ and CC_50_ values were calculated using non-linear regression with Graphpad Prism.

For the other CHIKV strains as well as for the additional alphaviruses, another variant of this protocol was used. For these viruses, Vero E6 cells were seeded in 2.5% supplemented Foetal Calf Serum (FCS) medium. The next day, two-fold serial dilutions of the compounds were added to the cells (25 μl/well). Fifteen minutes later, 25 μl of a virus mix containing the appropriate amount of viral stock dilution in medium was added to the 96-well plates. Cells were cultivated for 2 days and viral RNA was isolated as was described previously^12^.

### CHIKV quantitative reverse transcription-PCR (qRT-PCR)

CHIKV clinical isolates from Italy, Congo and Saint Martin were quantified by real time RT-PCR to determine viral RNA yield (SuperScript III Platinium one-step RT-PCR with Rox from Invitrogen), using CHIK-F2 TGGAATGGCTGGTTAACAAGATAA, CHIK-R2 CTCCGCGGACACCTAACG (except Congo strain: CHIK-R3 CTCCGCGGACACCTAWSG) and probe FAM-CTACTAAGAGAGTCACTTGGGTAG-MGB. Primers for amplification of other alphaviruses were described before^12^. For absolute quantification, standard curves were generated using 100-fold dilutions of T7 polymerase-generated RNA of known quantities for each virus. qRT-PCR was performed on a ABI 7900 HT Fast Real-Time PCR System, using 20 min at 50 °C and 3 min at 95 °C, followed by 40 cycles of 15 s at 95 °C and 1 min at 60 °C.

### Entry assay using CHIKV pseudoparticles

The protocol for the generation of CHIKV pseudotyped viruses was adapted from^15^. To generate the CHIKV glycoproteins expression vector, an expression cassette for E3/E2/E1 containing amino acids 262–1248 of the structural polyprotein was synthesized by RT-PCR on RNA isolated from the supernatant of CHIKV 899-infected cells. The expression cassette was subsequently cloned into vector pJET1.2 (Fermentas) by blunt-end ligation, after which it was cloned into the mammalian expression plasmid pcDNA3.1/Neo+ (Invitrogen) using NotI and XbaI. CHIKV pseudoparticles were produced essentially as described previously^32^ by transfecting HEK293T cells using a calcium phosphate based transfection protocol with 2 μg of the plasmid encoding the CHIKV envelope proteins, 2 μg of the packaging construct pBS-CMV-GagPol (a gift from Prof. Patrick Salmon (Addgene plasmid # 35614)) and 2 μg of a vector encoding luciferase as a marker protein (pGL3-Luc). Following 48 h of incubation at 37 °C the supernatant was collected and frozen at −20 °C. To evaluate the effect of MADTP molecules on CHIKVpp entry, Huh 7.5.1 cells were seeded at a density of 5000 cells/well in a white 96-well cell culture plate and allowed to adhere overnight at 37 °C. The next day, serial dilutions of the compounds and 100 μl of a 1/8 dilution of the CHIKVpp were added to the cells. As described before^15^, following 72 h of incubation at 37 °C, the cells were lysed and the luciferase activity was measured according to the manufacturer’s protocol (Luciferase Assay System, Promega).

### CHIKV translation assay

Translation of (non-replicating) CHIKV RNA into non-structural proteins was quantified basically as described^33^ except that Vero E6 cells were used (5 × 10^3^ per well) and replication-deficient CHIKV RNA (mutation in nsP4 active site) encoding a nsP3-Rluc fusion protein was transfected (100 ng per well). At 2.5, 5 and 7.5 h post transfection, cells were lysed in passive lysis buffer (Promega) and luciferase substrate was added according to the manufacturer’s instructions. Luciferase activity was measured in a GloMax 96 microplate luminometer (Promega).

### Metabolic labeling with ^3^H-uridine, *in vitro* RTC assay, denaturing agarose electrophoresis and in-gel hybridization

Actinomycin D (ActD; Sigma-Aldrich) was added to a final concentration of 5 μg/ml to 2.8 × 10^5^ CHIKV infected (MOI 5) or mock-infected Vero E6 cells in 12-well clusters at 5.5 h p.i. At 6 h p.i. viral RNA synthesis was labeled by adding 40 μCi of [^3^H]uridine to the medium. At 7 h p.i. total RNA was isolated and separated in denaturing agarose gels as described^19^. ^3^H-labeled RNA was visualized by fluorography. To correct for variations in loading, the gel was hybridized with a ^32^P-labeled oligonucleotide probe recognizing 18S ribosomal RNA. Detection of positive strand CHIKV RNA by in-gel hybridization with a ^32^P-labeled probe complementary to the 3′ end of the genome was done as described^19^. ^3^H-labeled RNA was quantified by scintillation counting and by fluorography and densitometry of scanned films. Hybridized gels were analyzed using PhosphorImager screens and a Typhoon 9410 imager (GE Healthcare), followed by quantification with Quantity One (BioRad). The RNA synthesizing activity of isolated CHIKV RTCs was determined by measuring incorporation of ^32^P-CTP in an *in vitro* assay that has been described previously^17^.

### Selection, purification and adaptation of MADTP-314 resistant virus isolates

To isolate MADTP-314-resistant virus variants, a 5-step protocol was used as previously published^12^. In a first step, Vero A cells were seeded in 100 μl of assay medium in 96-well microtiter plates at a density of 2.5 × 10^4^ cells/well and were allowed to adhere overnight. Subsequently, antiviral assays with MADTP-314 were set up using dilutions with different CCID_50_ of CHIKV 899 (ranging from 10 to 1000 CCID_50_). After 5 days of incubation, all assay wells were checked microscopically and quantitative data on cell survival were collected using the MTS/PMS method (see before). Based on these data, the lowest concentration of compound and the highest virus input at which complete and reproducible inhibition of virus-induced CPE was observed were selected. In a second step, three 96-well plates (a total of 144 assay wells) containing adherent Vero A cells were infected with the most optimal virus dilution (50 CCID_50_) and compound concentration (93 μM). After 5 days of incubation, most assay wells did not show any signs of virus-induced cell death. However, in some wells, virus break-through could be observed and the supernatant of the 3 wells with the most pronounced signs of virus-induced CPE was collected. These samples were purified in 6-fold by titration (1:5 dilution series) in the presence of 93 μM of MADTP-314. Three virus isolates (one from each original sample) were selected that produced the most pronounced signs of CPE in the presence of MADTP-314 at the lowest virus input possible. Subsequently, the resistant phenotype of the selected virus isolates was determined in comparison with wild-type virus. In parallel, the genotype was determined by full genome sequencing.

### Sequencing

Eight overlapping PCR amplicons were generated from viral RNAs previously extracted from the wild-type CHIKV 899 strain and from the putative-resistant virus isolates. Amplicons were generated by the OneStep RT-PCR kit (Qiagen), were gel purified and sequenced (BigDye^®^ Terminator v3.1 Cycle Sequencing Kit ABI) using primers that were published before^12^. The complete nucleotide sequences were assembled in ContigExpress (VNTI, Invitrogen) and the genomes of the resistant isolates were compared to the wild-type genome.

### Reverse genetics

Mutations were introduced in the infectious cDNA clone^19^ of CHIKV LS3 using the QuickChange mutagenesis kit (Agilent) according to the manufacturer’s instructions. The sequences of the used oligonucleotides are available upon request. Constructs were verified by sequencing using the BigDye Terminator Cycle Sequencing Kit v1.1 (Applied Biosystems) and a 3130 Genetic Analyzer automatic sequencer (Applied Biosystems). CHIKV was produced from these plasmids as described elsewhere^19^. Briefly, RNA was transcribed using the AmpliScribe T7 high yield transcription kit (Epicenter), the m^7^GpppA RNA cap structure analogue (NEB) and 0.7 μg of linearized template DNA. BHK-21 cells were electroporated with *in vitro* transcribed RNA using the Amaxa Nucleofector according to the manufacturer’s instructions. Infectious CHIKV from the supernatant of transfected cells (P0) harvested the day after transfection was used to grow working stocks (P1) of WT and mutant viruses on Vero E6 cells, which were used for further experiments. To confirm the presence of the introduced mutations (and absence of other mutations) the complete genomes of mutant viruses were sequenced by extracting viral RNA from virus stocks (P1) using the QIAamp Viral RNA Mini Kit (Qiagen). This RNA was used to generate four overlapping amplicons by reverse transcriptase PCR amplification which were sequenced completely.

### VEEV nsP1 enzyme assays

The protocols for VEEV nsP1 enzyme assays were adapted from^18^. In short, the codon optimized DNA encoding nsP1 of VEEV (strain P676, amino acid 1 to 535) was cloned into the expression vector pET28b (Novagen). Recombinant protein was produced in *E. coli* Rosetta pLysS (DE3) cells (Novagen), and then purified by IMAC chromatography. Site-directed mutagenesis was performed using the Quikchange site-directed mutagenesis Kit (Stratagene). The methyltransferase assay was carried out in a 20-μL reaction mixture, containing 50 mM Tris (pH 7.0), 2 mM DTT, 10 mM KCl, 2 mM GIDP, 330 nM S-adenosyl [methyl-^3^H] Methionine (83.1 Ci/mmol, PerkinElmer), 10 μM S-adenosylmethionine, 2 μM VEEV nsP1, 5% DMSO, and increasing concentrations of MADTPs. After incubation at 30 °C for 1 h, the reaction samples were loaded on DEAE-cellulose filter (PerkinElmer), and the filter was washed twice with 20 mM ammonium formate, once with H_2_O, and once with absolute ethanol. The filter was dried, and the radioactivity was measured by scintillation counting with SCINT BETAPLATE solution in a Wallac MicroBeta Trilux 1450 counter (PerkinElmer). The VEEV nsP1 guanylylation reactions were performed in 20 μL buffer containing 50 mM Tris (pH 7.0), 2 mM MgCl_2_, 2 mM DTT, 10 μM m^7^GTP, 100 μM AdoHcy and 2 μM nsP1 and incubated 1 h at 30 °C. Next, reactions were stopped, and subjected to western blot or ELISA using firstly anti-m_3_G/m^7^G-cap monoclonal antibody (Synaptic Systems) and secondary peroxidase-conjugated rabbit anti-mouse antibody (Sigma, #A9044). For the Western blot assay, the immunoreactive proteins were detected using the Pierce ECL Western Blotting Substrate (ThermoScientific) and a Kodak Image Station 4000MM Pro (Carestream Health, Inc.). The signal was quantified using ImageJ software.

## Additional Information

**How to cite this article**: Delang, L. *et al*. The viral capping enzyme nsP1: a novel target for the inhibition of chikungunya virus infection. *Sci. Rep.* **6**, 31819; doi: 10.1038/srep31819 (2016).

## Supplementary Material

Supplementary Information

## Figures and Tables

**Table 1:** *In vitro* antiviral activity of analogs in the MADTP series against CHIKV and VEEV.

Species	Virus (strain)	MADTP-314	MADTP-372	MADTP-393
CHIKV	*899 (lab)*	26 ± 11^a^	2.6 ± 1.0^a^	18 ± 6.4^a^
	*Venturini (Italy 2008)*	26 ± 2^b^	1.4 ± 0.01^b^	8.2 ± 2.2^b^
	*Congo 95 (2011)*	6.4 ± 0.1^b^	0.75 ± 0.4^b^	1.4 ± 0.3^b^
VEEV	*TC83*	>40^b^	6.8^b^	35 ± 10^b^

^a^CPE reduction, ^b^viral RNA reduction. VEEV: Venezuelan equine encephalitis virus. Data shown are average values ± SD of at least two independent experiments. CC_50_ values for MADTP-314, MADTP-372 and MADTP-393 on Vero cells are >743 μM, >668 μM and 57 ± 28 μM, respectively.

**Table 2:** *In vitro* anti-CHIKV activity of MADTP molecules against WT and resistant CHIKV variants.

EC_50_ (μM)	Resistance selection		Reverse genetics	
	WT 899	MADTP-314^res^ variant 3	WT LS3	LS3-P34S
MADTP-314	26 ± 11	283 ± 45	40 ± 5.0	>500
MADTP-372	2.6 ± 1.0	255 ± 16	4.9 ± 0.6	>50
MADTP-393	18 ± 6.4	193 ± 14	15 ± 0.8	>50
Chloroquine	18 ± 11	20 ± 4.7	n.d.	n.d.
T-705	20 ± 2.7	34 ± 5.7	n.d.	n.d.

n.d.: not determined.

Data shown are average values ± SD of at least two independent experiments.

## References

[b1] Powers A. M. . Evolutionary relationships and systematics of the alphaviruses. J. Virol. 75, 10118–10131 (2001). 11581380 10.1128/JVI.75.21.10118-10131.2001 PMC114586

[b2] Chen L. H. & Wilson M. E. Dengue and chikungunya in travelers. Current Opinion in Infectious Diseases 25, 523–529 (2012). 22825287 10.1097/QCO.0b013e328356ffd5

[b3] Tilston N., Skelly C. & Weinstein P. Pan-European Chikungunya surveillance: designing risk stratified surveillance zones. Int. J. Health Geogr. 8, 61 (2009). 19878588 10.1186/1476-072X-8-61 PMC2776014

[b4] Leparc-Goffart I., Nougairede A., Cassadou S., Prat C. & De Lamballerie X. Chikungunya in the Americas. The Lancet 383, 514 (2014). 10.1016/S0140-6736(14)60185-9 24506907

[b5] Hoz J. M. D. La . Fatal cases of Chikungunya virus infection in Colombia: Diagnostic and treatment challenges. J. Clin. Virol. 69, 27–29 (2015). 26209372 10.1016/j.jcv.2015.05.021

[b6] Torres J. R. . Chikungunya fever: Atypical and lethal cases in the Western hemisphere. IDCases 2, 6–10 (2015). 26793440 10.1016/j.idcr.2014.12.002 PMC4672604

[b7] Singh S. K. & Unni S. K. Chikungunya virus: Host pathogen interaction. Rev. Med. Virol. 21, 78–88 (2011). 21412934 10.1002/rmv.681

[b8] Schilte C. . Chikungunya virus-associated long-term arthralgia: a 36-month prospective longitudinal study. PLoS Negl. Trop. Dis. 7, e2137 (2013). 23556021 10.1371/journal.pntd.0002137 PMC3605278

[b9] Khan M., Santhosh S. R., Tiwari M., Lakshmana Rao P. V. & Parida M. Assessment of *in vitro* prophylactic and therapeutic efficacy of chloroquine against Chikungunya virus in Vero cells. J. Med. Virol. 82, 817–824 (2010). 20336760 10.1002/jmv.21663 PMC7166494

[b10] Brighton S. W. Chloroquine phosphate treatment of chronic Chikungunya arthritis. An open pilot study. South African Med. J. 66, 217–218 (1984). 6087474

[b11] De Lamballerie X. . On chikungunya acute infection and chloroquine treatment. Vector borne zoonotic Dis. 8, 837–839 (2008). 18620511 10.1089/vbz.2008.0049

[b12] Delang L. . Mutations in the chikungunya virus non-structural proteins cause resistance to favipiravir (T-705), a broad-spectrum antiviral. J. Antimicrob. Chemother. 69, 2770–2784 (2014). 24951535 10.1093/jac/dku209

[b13] Abdelnabi R., Neyts J. & Delang L. Towards antivirals against the chikungunya virus. Antiviral Res. 121, 59–68 (2015). 26119058 10.1016/j.antiviral.2015.06.017 PMC7113767

[b14] Gigante A. . Identification of [1,2,3]triazolo[4,5-d]pyrimidin-7(6H)-ones as novel inhibitors of Chikungunya virus replication. J. Med. Chem. 57, 4000–4008 (2014). 24800626 10.1021/jm401844c

[b15] Salvador B., Zhou Y., Michault A., Muench M. O. & Simmons G. Characterization of Chikungunya pseudotyped viruses: Identification of refractory cell lines and demonstration of cellular tropism differences mediated by mutations in E1 glycoprotein. Virology 393, 33–41 (2009). 19692105 10.1016/j.virol.2009.07.013 PMC2760448

[b16] Delogu I. . *In vitro* antiviral activity of arbidol against Chikungunya virus and characteristics of a selected resistant mutant. Antiviral Res. 90, 99–107 (2011). 21440006 10.1016/j.antiviral.2011.03.182

[b17] Albulescu I. C., Tas A., Scholte F. E. M., Snijder E. J. & van Hemert M. J. An *in vitro* assay to study chikungunya virus RNA synthesis and the mode of action of inhibitors. J. Gen. Virol. 95, 2683–2692 (2014). 25135884 10.1099/vir.0.069690-0

[b18] Li C. . mRNA capping by Venezuelan Equine Encephalitis Virus nsP1: Functional characterization and implication for antiviral research. J. Virol. 89, 8292–8303 (2015). 26041283 10.1128/JVI.00599-15 PMC4524220

[b19] Scholte F. E. M. . Characterization of synthetic Chikungunya viruses based on the consensus sequence of recent E1-226V isolates. PLoS One 8, e71047 (2013). 23936484 10.1371/journal.pone.0071047 PMC3731263

[b20] Decroly E., Ferron F., Lescar J. & Canard B. Conventional and unconventional mechanisms for capping viral mRNA. Nat. Rev. Microbiol. 10, 51–65 (2012). 22138959 10.1038/nrmicro2675 PMC7097100

[b21] Ahola T., Laakkonen P., Vihinen H. & Kääriäinen L. Critical residues of Semliki Forest virus RNA capping enzyme involved in methyltransferase and guanylyltransferase-like activities. J. Virol. 71, 392–397 (1997). 8985362 10.1128/jvi.71.1.392-397.1997 PMC191063

[b22] Lin H. Y., Yu C. Y., Hsu Y. H. & Meng M. Functional analysis of the conserved histidine residue of Bamboo mosaic virus capping enzyme in the activity for the formation of the covalent enzyme-m7GMP intermediate. FEBS Lett. 586, 2326–2331 (2012). 22641040 10.1016/j.febslet.2012.05.024

[b23] Vasiljeva L., Merits A., Auvinen P. & Kääriäinen L. Identification of a novel function of the Alphavirus capping apparatus. RNA 5′-triphosphatase activity of Nsp2. J. Biol. Chem. 275, 17281–17287 (2000). 10748213 10.1074/jbc.M910340199

[b24] Karpe Y. A., Aher P. P. & Lole K. S. NTPase and 5′-RNA triphosphatase activities of chikungunya virus nsP2 protein. PLoS One 6, e22336 (2011). 21811589 10.1371/journal.pone.0022336 PMC3139623

[b25] DuBois R. M. . Structural and Biochemical Basis for Development of Influenza Virus Inhibitors Targeting the PA Endonuclease. PLoS Pathog. 8, e1002830 (2012). 22876176 10.1371/journal.ppat.1002830 PMC3410894

[b26] Ferron F., Decroly E., Selisko B. & Canard B. The viral RNA capping machinery as a target for antiviral drugs. Antiviral Res. 96, 21–31 (2012). 22841701 10.1016/j.antiviral.2012.07.007 PMC7114304

[b27] Balzarini J., De Clercq E., Serafinowski P., Dorland E. & Harrap K. R. Synthesis and antiviral activity of some new S-adenosyl-L-homocysteine derivatives. J. Med. Chem. 35, 4576–4583 (1992). 1335077 10.1021/jm00102a010

[b28] Ahola T. & Karlin D. G. Sequence analysis reveals a conserved extension in the capping enzyme of the alphavirus supergroup, and a homologous domain in nodaviruses. Biol. Direct 10, 1–21 (2015). 25886938 10.1186/s13062-015-0050-0 PMC4392871

[b29] Sokoloski K. J. . Noncapped alphavirus genomic RNAs and their role during infection. J. Virol. 89, 6080–6092 (2015). 25833042 10.1128/JVI.00553-15 PMC4442418

[b30] Panning M., Grywna K., Van Esbroeck M., Emmerich P. & Drosten C. Chikungunya fever in travelers returning to Europe from the Indian Ocean Region, 2006. Emerg. Infect. Dis. 14, 416–422 (2008). 18325256 10.3201/eid1403.070906 PMC2570846

[b31] Tan L. Van . Me Tri virus: A Semliki Forest virus strain from Vietnam? J. Gen. Virol. 89, 2132–2135 (2008). 18753222 10.1099/vir.0.2008/002121-0

[b32] Lavillette D. . Hepatitis C virus glycoproteins mediate low pH-dependent membrane fusion with liposomes. J. Biol. Chem. 281, 3909–3917 (2006). 16356932 10.1074/jbc.M509747200

[b33] Scholte F. E. M. . Stress granule components G3BP1 and G3BP2 play a proviral role early in Chikungunya virus replication. J. Virol. 89, 4457–4469 (2015). 25653451 10.1128/JVI.03612-14 PMC4442398

[b34] Metz S. W. . Functional processing and secretion of Chikungunya virus E1 and E2 glycoproteins in insect cells. Virol. J. 8, 353 (2011). 21762510 10.1186/1743-422X-8-353 PMC3162542

