# Supplementary material for: The viral capping enzyme nsP1: a novel target for the inhibition of chikungunya virus infection
Source: Sci Rep. 2016 Aug 22;6:31819. doi: 10.1038/srep31819 (PMC4992889; doi:10.1038/srep31819)
Supplement: Supplementary Information [file srep31819-s1.pdf]

Supplemental Information

The viral capping enzyme nsP1: a novel target for the inhibition of chikungunya virus infection

L. Delang[1], C. Li[2,3], A. Tas[4], G. Quérat[5], I. C. Albulescu[4], T. De Burghgraeve[1], N. A. Segura Guerrero[1], A. Gigante[6], G. Piorkowski[5], E. Decroly[2,3], D. Jochmans[1], B. Canard[2,3], E. J. Snijder[4], M. J. Pérez-Pérez[6], M. J. van Hemert[4], B. Coutard[2,3], P. Leyssen[#1], J. Neyts*[#1].

Supplemental Figure 1. Multiple sequence alignment of nsP1 amino acid sequences of alphaviruses.

A

```
CHIKV      --MDPVYYVDIDADSAFLKALQRAYPMFEVEPRQVTPNDHANARAFSHLAIKLIEQEIDPD 58
ONNV       --MDSVYYVDIDADSAFLKALQQAYPMFEVEPKQVTPNDHANARAFSHLAIKLIEQEIDPD 58
RRV        ---MKVTVDVEADSPFLKALQKAFPAFEVESQQVTPNDHANARAFSHLATKLIEQEVPAN 57
SFV        -MAAKVHVDIEADSPFIKSLQKAFPSFEVESLQVTPNDHANARAFSHLATKLIEQETDKD 59
SINV       MEKPVVNVDVDPQSPFVVQLQKSFPQFEVVAQQVTPNDHANARAFSHLASKLIELEVPTT 60
WEEV       --MERIHVDLDADSPYVKSLQRTFPQFEIEARQVTDNDHANARAFSHVATKLIESEVDRD 58
EEEV       --MEKVHVDLDADSPFVKSLQRCFPHFEIEATQVTDNDHANARAFSHLATKLIEGEVDTD 58
VEEV       --MEKVHVDIEEDSPFLRALQRSFPQFEVEAKQVTDNDHANARAFSHLASKLIETEVDPS 58
                :  **::  :*.::   **:  :* **:  .  ***  ***********:*  ****  *
```

B

```
899            MDPVYYVDIDADSAFLKALQRAYPMFEVEPRQVTPNDHANARAFSHLAIKLIEQEIDPDST
Venturini      MDPVYYVDIDADSAFLKALQRAYPMFEVEPRQVTPNDHANARAFSHLAIKLIEQEIDPDST
Congo          MDPVYYVDIDADSAFLKALQRAYPMFEVESRQVTPNDHANARAFSHLAIKLIEQEIDPDST
LS3            MDPVYYVDIDADSAFLKALQRAYPMFEVEPRQVTPNDHANARAFSHLAIKLIEQEIDPDST
               *****************************.*******************************

899            ILDIGSAPARRMMSDRKYHCVCPMRSAEDPERLANYARKLASAAGKVLDRNISGKIGDLQ
Venturini      ILDIGSAPARRMMSDRKYHCVCPMRSAEDPERLANYARKLASAAGKVLDRNISGKIGDLQ
Congo          ILDIGSAPARRMMSERKYHCVCPMRSAEDPERLANYARKLASAAGKVLDRNISGKIGDLQ
LS3            ILDIGSAPARRMMSDRKYHCVCPMRSAEDPERLANYARKLASAAGKVLDRNISGKIGDLQ
               **************:*********************************************

899            AVMAVPDTETPTFCLHTDVSCRQRADVAIYQDVYAVHAPTSLYHQAIKGVRVAYWVGFDT
Venturini      AVMAVPDKETPTFCLHTDVSCRQRADVAIYQDVYAVHAPTSLYHQAIKGVQVAYWVGFDT
Congo          AVMAVPDTETPTFCLHTDVSCRQRADVAIYQDVYAIHAPTSLYHQAIKGVRVAYWVGFDT
LS3            AVMAVPDKETPTFCLHTDVSCRQRADVAIYQDVYAVHAPTSLYHQAIKGVRVAYWVGFDT
               *******.***************************:**************.*********

899            TPFMYNAMAGAYPSYSTNWADEQVLKAKNIGLCSTDLTEGRRGKLSIMRGKKLKPCDRVL
Venturini      TPFMYNAMAGAYPSYSTNWADEQVLKAKNIGLCSTDLTEGRRGKLSIMRGKKLKPCDRVL
Congo          TPFMYNAMAGAYPSYSTNWADEQVLKAKNIGLCSTDLTEGRRGKLSIMRGKKLKPCDRVL
LS3            TPFMYNAMAGAYPSYSTNWADEQVLKAKNIGLCSTDLTEGRRGKLSIMRGKKLKPCDRVL
               ************************************************************

899            FSVGSTLYPESRKLLKSWHLPSVFHLKGKLSFTCRCDTVVSCEGYVVKRITMSPGLYGKT
Venturini      FSVGSTLYPESRKLLKSWHLPSVFHLKGKLSFTCRCDTVVSCEGYVVKRITMSPGLYGKT
Congo          FSVGSTLYPESRKLLKSWHLPSVFHLKGKLSFTCRCDTVVSCEGYVVKRITMSPGLYGKT
LS3            FSVGSTLYPESRKLLKSWHLPSVFHLKGKLSFTCRCDTVVSCEGYVVKRITMSPGLYGKT
               ************************************************************

899            TGYAVTHHADGFLMCKTTDTVDGERVSFSVCTYVPATICDQMTGILATEVTPEDAQKLLV
Venturini      TGYAVTHHADGFLMCKTTDTVDGERVSFSVCTYVPATICDQMTGILATEVTPEDAQKLLV
Congo          TGYAVTHHADGFLMCKTTDTVDGERVSFSVCTYVPATICDQMTGILATEVTPEDAQKLLV
LS3            TGYAVTHHADGFLMCKTTDTVDGERVSFSVCTYVPATICDQMTGILATEVTPEDAQKLLV
               ************************************************************

899            GLNQRIVVNGRTQRNTNTMKNYLLPVVAQALSKWAKECRKDMEDEKLLGVRERTLTCCCL
Venturini      GLNQRIVVNGRTQRNMNTMKNYLLPVVAQAFSKWAKECRKDMEDEKLLGVRERTLTCCCL
Congo          GLNQRIVVNGRTQRNTNTMKNYLLPVVAQAFSKWAKECRKDMEDEKLLGVRERTLTCCCL
LS3            GLNQRIVVNGRTQRNMNTMKNYLLPVVAQAFSKWAKECRKDMEDEKLLGVRERTLTCCCL
               *************** **************:*****************************

899            WAFKKQKTHTVYKRPDTQSIQKVQAEFDSFVVPSLWSSGLSIPLRTRIKWLLSKVPKTDL
Venturini      WAFKKQKTHTVYKRPDTQSIQKVQAEFDSFVVPSLWSSGLSIPLRTRIKWLLSKVPKTDL
Congo          WAFKKQKTHTVYKRPDTQSIQKVQAEFDSFVVPSLWSSGLSIPLRTRIKWLLSKVPKTDL
LS3            WAFKKQKTHTVYKRPDTQSIQKVQAEFDSFVVPSLWSSGLSIPLRTRIKWLLSKVPKTDL
               ************************************************************

899            IPYSGDAREARDAEKEAEEEREAELTREALPPLQAAQEDVQVEIDVEQLEDRAGA
Venturini      IPYSGDAREARDAEKEAEEEREAELTREALPPLQAAQEDVQVEIDVEQLEDRAGA
Congo          IPYSGDTQEARDAEKEAEEEREAELTRVALPPLQAAQEDVQVEIDVEQLEDRAGA
LS3            IPYSGDAREARDAEKEAEEEREAELTREALPPLQAAQEDVQVEIDVEQLEDRAGA
               ******:.******************** **************************
```

A) The nsP1 amino acid sequences of the CHIKV strain 899 (ACV88658.1), ONNV (NP_041254.1), RRV (NP_062879.1), SFV (NP_463457.1), SINV (NP_062888.1), WEEV (NP_640330.1), EEV (NP_632021.2), and VEEV (AAY16003.1) were aligned using the ClustalW2 server (http://www.ebi.ac.uk/Tools/msa/clustalw2/) (1). Conserved amino acid sequences are shown in grey. The residues corresponding to P34 in CHIKV are highlighted in yellow as well as the corresponding D34 in the other alphaviruses. The conserved histidine of the methyltransferase motif is shown in green. B) The nsP1 sequences of the CHIKV strain 899 (ACV88658.1), clinical isolates Venturini (Italy 2008) and Congo 95 (2011) and CHIKV infectious clone LS3 (AIK21921.1) were aligned using the Muscle software (http://www.ebi.ac.uk/Tools/msa/muscle/). The residues corresponding to P34 in CHIKV 899 are highlighted in green. The residues of CHIKV Venturini, Congo and LS3 that differ from CHIKV 899 are highlighted in yellow, blue and pink, respectively.

Supplemental Figure 2. Production and functional comparison of CHIKV and VEEV nsP1.

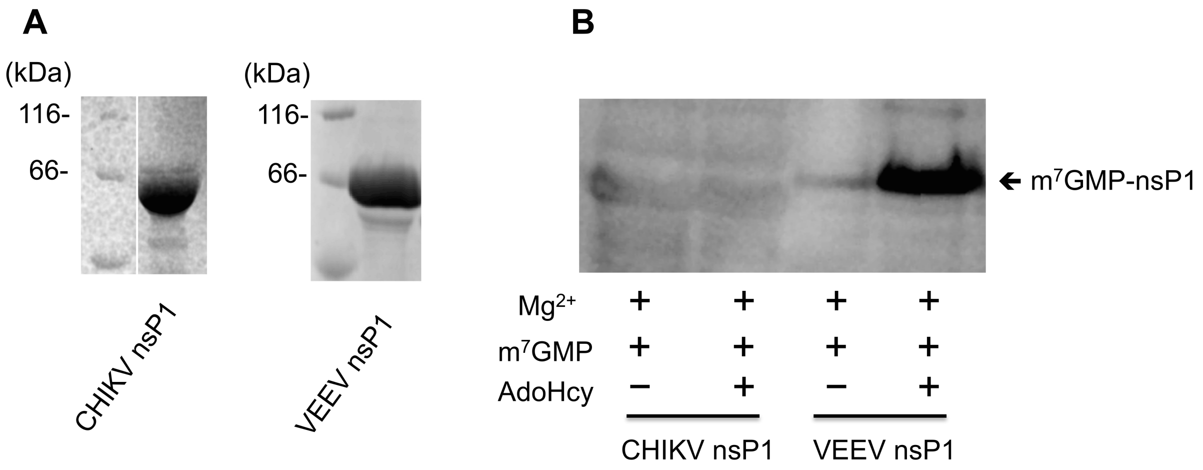

A) Coomassie blue-stained SDS-PAGE gel showing the produced CHIKV and VEEV nsP1 proteins. B) Western blotting showing the formation of m^7GMP-nsP1 complex by CHIKV and VEEV nsP1.

Supplemental Table 1. Mutations identified in the MADTP-314-resistant CHIKV isolates.

Gene	WT codon	WT AA	Mutant codon			Mutant AA		
			**c1**	**c2**	**c3**	**c1**	**c2**	**c3**
**nsP1**	GAC	D_9	GAC	GAC	**T**AC	D_9	D_9	**Y**$_9$
	CAA	Q_{19}	CAA	CAA	C**G**A	Q_{19}	Q_{19}	**R**$_{19}$
	CCG	P_{34}	**T**CG	**T**CG	**T**CG	**S**$_{34}$	**S**$_{34}$	**S**$_{34}$
**nsP3**	GCC	A_{458}	**T/G**CC	n.d.	n.d.	**S/A**$_{458}$	n.d.	n.d.
**E1**	GTG	V_6	G**A/T**G	n.d.	n.d.	**E/V**$_6$	n.d.	n.d.

n.d.: not determined; c: independently selected resistant clone.

References

1. McWilliam H, et al. (2013) Analysis Tool Web Services from the EMBL-EBI. *Nucleic Acids Res* 41:W597–600.
